# Effect of combined oral contraceptive on cardiorespiratory function and immune activation in premenopausal women involved in exercise: A systematic review protocol

**DOI:** 10.1371/journal.pone.0298429

**Published:** 2024-02-23

**Authors:** Oyesanmi A. Fabunmi, Phiwayinkosi V. Dludla, Bongani B. Nkambule

**Affiliations:** 1 School of Laboratory Medicine and Medical Sciences (SLMMS), University of KwaZulu-Natal, Durban, South Africa; 2 Health-awareness, Exercise and Cardio-immunologic Research Unit (HECIRU), Department of Physiology, College of Medicine, Ekiti State University, Ado-Ekiti, Nigeria; 3 Cochrane South Africa, South African Medical Research Council, Tygerberg, South Africa; 4 Department of Biochemistry and Microbiology, University of Zululand, KwaDlangezwa, South Africa; Jatiya Kabi Kazi Nazrul Islam University, BANGLADESH

## Abstract

**Background:**

The use of combined oral contraceptive (COC) is common among women of reproductive age despite the potential risk of them developing thrombotic events. There is a need to understand how COC affects cardiorespiratory function and markers of immune activation in premenopausal women involved in exercise. This highlights a need for a systematic review to enhance our understanding of how the use of COC affects cardiovascular health in premenopausal women subjected to exercise.

**Method:**

This systematic review protocol was prepared following the Preferred Reporting Items for Systematic Reviews and Meta-Analyses Protocols (PRISMA-P) 2015 statement. An extensive search of relevant literature by two independent reviewers will be conducted through the EBSCOhost interface to access databases such as MEDLINE, EMBASE, and CINAHL. Other health sources, including Cochrane CENTRAL, unpublished studies and grey literature, will also be searched. The search will include all studies that report the effect of COC on essential parameters of cardiorespiratory function and markers of immune activation in premenopausal women involved in exercise. All included studies will be appraised using appraisal tools, while appropriate extraction tools will be used for data extraction. Where possible, eligible studies will be pooled for meta-analysis. If statistical pooling is not feasible, our findings will be presented in a narrative format. The certainty of evidence will be assessed using the Grading of Recommendations, Assessment, Development and Evaluation Assessment (GRADE) tool.

**Trial registration:**

**PROSPERO registration number:** CRD42021265257.

## Introduction

Regular exercise is associated with numerous physiological, metabolic, and psychological benefits [1]. Combined oral contraceptives (COCs), or progestogen-only pills, remain one of the most commonly used modern methods of contraception among premenopausal women [2]. Briefly, the premenopausal phase is based on a bleeding pattern with regular menstrual cycles in women of reproductive age [3]. Evidence suggests an association between exogenous and endogenous hormones on exercise performance due to their profound effects on respiratory function [4–6]. For instance, the use of COC is associated with impaired maximal oxygen uptake ($\dot{V}O_{2}$ max) in trained premenopausal women [7]. In contrast, other studies reported that using COC does not affect $\dot{V}O_{2}$ max during the pill cycle in some women [8, 9]. The type of COC, variations in the menstrual cycle phase and the methodological approach adopted by the studies among other factors appears to influence the observed variations in the reported outcome [9].

Briefly, $\dot{V}O_{2}$ max is regarded as an index of exercise performance or cardiorespiratory fitness in an individual [4, 5]. It also serves as the gold standard for assessing the combined performance of the respiratory, cardiovascular, and muscular systems during oxygen translocation, absorption, and utilization within the active muscle mitochondria [10]. It is unclear whether hormonal fluctuation also affects other parameters of ventilatory efficiency such as minute ventilation (VE), respiratory exchange ratio (RER) and VCO_2_, which could also be of prognostic benefit in evaluating exercise performance.

RER for instance is associated with substrate utilization [11] and alteration in the basal metabolic rate (BMR) in women of reproductive age that are performing exercise [12]. Substrate utilization depict how energy expenditure is derived from fat and carbohydrates in exercise condition [13]. Although, controversy persist on the extent at which COC influences substrate utilization from basal level into exercise condition in premenopausal women. While some studies reported an association between COC and changes in the lipid profile and glucose metabolism [14–16], other several previous studies reported no significant change in some of these measured metabolic parameters [17–20].

Of note, substrate utilization increases rapidly in the heart vasculature during exercise [21]. In the vasculature, estrogen may antagonize the vasoactive effect of progesterone thereby increasing the rate of oxygen delivery to the tissue and increasing the VO_2_ max [22–24]. Despite the favourable effect of estrogen on metabolic rate and the vasculature, it is unclear whether the intrinsic androgenicity of the progestin component of COC has a role to play in the rate of substrate utilization that extend into the vasculature [20, 25, 26]. Another major concern of interest among female athletes is the issue of increased weight gain that is linked to the metabolic effects of COC [27–29]. While the effect of COC on weight gain remain controversial [30], weight gain probably remain one of the most relevant physiologic variations among young female athletes, during which a slight alteration in their body configuration may influence their physical and psychological performance [31].

Again, an inverse correlation exist between biomarkers of inflammation and cardiorespiratory fitness in certain individuals exposed to exercise [32, 33]. The extent to which gender differences and cyclical variations of sex hormones influence immunological responses during exercise is unclear [34–36]. In fact, available evidence regarding the effect of COC on the markers of immune activation [37–41] especially in exercise condition are limited and inconsistent. For instance, COC was shown to increase the levels of interleukin (IL)-1ra, IL-8 and C-reactive protein (CRP) during exercise [42, 43]. In contrast, some studies reported decrease in the levels of inflammatory cytokines such as IL-6 in COC users compared to naturally cycling women [35]. While in other studies, COC did not alter the levels of IL-6 and other related cytokines during exercise [43, 44]. The duration and intensity of exercise among other factors like age appears to play a major role in regulating immune function [45, 46]. However, it is unclear whether the type of COC and duration of use are associated with changes in immune response in different population undergoing the same or different exercise regimen.

It is noteworthy that previous reviews have been conducted to evaluate the effect of oral contraceptive use on exercise performance in women of reproductive age [6, 47]. However, non-randomised observational studies were the main source of data used in Elliott-Sale et al., 2020 investigation which was deemed a significant constraint that resulted in an inconclusive outcome [6]. Whereas a recent study by Schumpf et al., 2023 only focused on the mean relative $\dot{V}O_{2}$ max as a measure of exercise performance without considering other parameters such as immune activation that are also valuable during comprehensive assessment of cardiorespiratory fitness [47]. Although unique for specifically targeting cardiorespiratory hemodynamics and immune cell activation in exercising individuals, the proposed systematic review further build on the synthesis of evidence already published clarifying the status of cardiovascular health in response to the use of oral contraceptives in women of reproductive age [48].

Thus, the proposed systematic review and meta-analysis aim to broadly synthesise the available evidence regarding the effect of COC on cardiorespiratory function and makers of immune activation in premenopausal women who are involved in exercise. Further, we will assess if the reported outcomes are associated specifically with the type of COC and duration of use. Hence, knowing the magnitude at which individual COC formulations affects cardiorespiratory function and the markers of immune activation in exercise condition may help provide awareness and guidance when deciding on the choice of available COC during consultation and follow up. In addition, it will also provide guidance during planning of future experimental studies which will involve digging the various mechanism of how COC regulates the cardiorespiratory and immune function.

### Review question

1. What is the effect of COC intake on cardio-respiratory and immune function in premenopausal women that are involved in exercise compared to those not taking COC.

## Methods

### Study registration

The systemic review protocol was prepared using the preferred reporting items for systemic review and meta-analysis protocol (PRISMA-P) (S1 Checklist). This protocol was registered with the International Prospective Register of Systematic Reviews: Prospero (CRD42021265257). The JBI guideline and methodology regarding the systematic review of effectiveness was also considered during the course of the review [49].

### Eligibility criteria

#### Types of studies

The type of studies that will be included in our review are mainly experimental studies such as cross-sectional, cohort, and randomized control trials. We will not include qualitative, delphi or q methodology study types. Reviews, books, and letters to editors will also not be considered.

#### Participants

Participants of included studies will be premenopausal women between 15–50 years. Participants of the included studies who are smokers will not be included in our systematic review.

#### Intervention

The systematic review will include studies that report using all types of COC (first, second, third and newer-generation types). Our intervention of interest (COC) is the most commonly used form of contraception among premenopausal women due to its safety profile and accessibility. Aside from the use of COCs for birth control, COCs are also used as an alternative to alleviate symptomatic side effects such as cramps/pain, bloating and headaches that are associated with eumenorrheic menstrual cycle and to prevent unpredictable menstruation in athletic women [2, 50].

#### Comparator

This will include study participants who are not using COC but are physically active or involved in exercise.

#### Outcomes

Any study identified that reported any of the related outcomes of interest will be considered. The outcome of interest will include the following:

#### Primary and surrogate outcome

- The cardiorespiratory function with be determined by the VO_2_max, heart rate, VCO_2_, and VE.
- The change in immune function will be assessed by measuring the levels of IL-1β and IL-8.
- The changes in metabolic rate and body composition will be assessed through respiratory exchange ratio (RER), blood lactate concentration, body mass index (BMI) and the waist-hip ratio.

### Search strategy and information sources

At the initial stage, we searched MEDLINE and CINAHL to identify articles that are relevant to the topic without language restriction on August 15, 2023. We then developed a full search strategy for MEDLINE (S1 File) using the text words contained in the titles and abstracts of relevant articles as well as the index terms used to describe the articles. Subsequently, a modified full search strategy including the keywords and index terms, or medical subheadings (MeSH) will be adopted for each information source that will be included in the main review [51, 52]. The keywords and MeSH terms will include “oral contraceptive pills”, “birth control pills”, “oral contraceptives”, “contraceptives”, “exercise”, “physical activity” “cardiorespiratory fitness”, “immune activation”, and “premenopausal women”. In furtherance, we will scan the reference list of the selected studies and undertake a forward citation tracking via Google scholar to identify relevant literature. Studies published from inception till date from all databases will be included to capture all relevant evidence without language limitation; however, only studies that could be translated to English (using Google translate) will be included as part of this review. We will conduct a broad search through the EBSCOhost and Ovid interface to access the following databases: MEDLINE, EMBASE and Cumulative Index to Nursing and Allied Health Literature (CINAHL) from inception till date. Furthermore, the Cochrane Central Register of Clinical trials (CENTRAL) will also be searched to augment relevant source of information. OpenGrey (System Information on Grey Literature in Europe) (www.open.eu), ClinicalTrials.gov will be searched for relevant unpublished studies and grey literature.

### Study selection

At the Initial stage, the titles, abstracts, keywords, and synonyms will be used to screen the studies and identify the relevant articles. Two separate authors will screen the studies to ensure consistency in determining study eligibility. This approach aims to enhance objectivity, minimize errors, and promote accuracy in the screening process. Should discrepancies arise, the third independent reviewer will be consulted. Extracted data items will be sorted, including archiving of relevant and excluded studies with reasons via the Mendeley desktop reference manager version 1.19.4 (Mendeley Ltd., Elsevier, Netherlands). Importantly, we will screen the reference lists of all included studies to be sure no relevant studies are left out. The PRISMA flow diagram will be used to present the full results of our search and study selection as well as the inclusion process in the final systematic review [53]. Each study meeting the inclusion criteria will undergo data collection and a risk of bias assessment. The appropriate appraisal tools will be employed for this evaluation [54, 55].

### Assessment of methodological quality

Two independent reviewers will assess the methodological quality of eligible studies using the applicable appraisal checklist from JBI [54]. We will reach out to the authors of the papers to request any missing or supplementary data necessary for clarification, if needed. We will resolve any sort of disagreement through discussion or with a third reviewer (BBN). Irrespective of the methodological quality outcome, all studies will undergo data extraction and synthesis, where applicable. A summary of the appraisal results will be presented in tabular form and incorporated into the review.

### Data extraction

The retrieval of pertinent data items will be executed using the structured and standardized JBI data extraction tool [56, 57]. During the data extraction from selected studies, OAF and PVD will independently handle data entry to minimize errors. In cases of discrepancies, BBN will be consulted to facilitate consensus. The following information will be extracted from the selected studies: author and year of publication, country, population details (sample size), study design, specifics of contraceptive usage (types, dosage, and duration), cardiorespiratory function index (including HR, VO_2_max, VCO2, VE), markers of immune activation (such as IL-1β, IL-8), and parameters related to metabolic changes and body composition (RER, blood lactate concentration, and waist-hip ratio) for both oral contraceptive users and non-users. In cases of insufficient data, efforts will be made to contact the primary authors of the studies to acquire necessary information.

### Data synthesis

Where possible, studies will be pooled in a statistical analysis using Review Manager (RevMan) version 5.3 (The Nordic Cochrane Centre, The Cochrane Collaboration, 2014). Firstly, we will perform an assessment of clinical and methodological heterogeneity, followed by an evaluation of statistical heterogeneity. The Chi-squared (*X*^*2*^) and *I*^*2*^ statistic tests will be employed to ascertain the extent of heterogeneity across all the included studies. A value *I*^*2*^ >50 will be interpreted as indicating moderate or substantial heterogeneity [58, 59]. In cases where there are similarities among the included studies concerning participants, interventions, comparisons, and outcomes, a fixed-effect meta-analysis will be conducted. Conversely, if the studies exhibit a substantial level of heterogeneity, a random-effects model will be employed. Furthermore, to investigate the reason behind heterogeneity within the included studies, we will undertake a subgroup analysis or sensitivity analysis. Meta-regression will be conducted based on factors the overall risk of bias, study design, location of study, dosage, and duration of contraceptive usage, intensity, and duration of exercise. These analyses aim to provide insights into potential sources of variation among the studies. In instances where statistical grouping is not feasible, we will present the synthesis in a narrative format, incorporating tables and figures where applicable to effectively convey the data. Additionally, when there are 10 or more studies included in a meta-analysis, we will assess publication bias through the use of a funnel plot. To test for the asymmetry of the funnel plot, we will employ the appropriate statistical test available, such as the Egger test, Begg test, or Harbord test. These analyses contribute to evaluating potential publication bias in the included studies.

### Evaluating the certainty of evidence

To evaluate the certainty of the findings, we will employ a systematic approach, using the GRADE (Grading of Recommendations, Assessment, Development, and Evaluation) framework [60, 61]. This involves considering factors such as risk of bias, consistency of results, directness of evidence, precision, and publication bias. We will use GRADEPro GDT (McMaster University, ON, Canada) to create a Summary of Findings (SoF) table. The SoF table will include crucial information such as absolute risks for both treatment and control groups, estimates of relative risk, and a quality ranking of the evidence. The SoF will cover outcomes related to cardiorespiratory function, markers of immune activation, and parameters of metabolic changes and body composition. The GRADE approach will provide the basis for making recommendations and conclusions.

## Discussion

Women’s exercise participation, ranging from physical activity to competitive sports, has increased within the past few decades [62]. The cyclical variations in endogenous sex hormones (progesterone and estrogen) throughout the menstrual period have been proposed to potentially influence performance [63]. Other previous studies have evaluated the effect of hormonal contraceptive on exercise performance [6, 47]. However, the possible mechanisms by which COC influence exercise performance in women of reproductive age remain controversial and unclear due to differential methodological approach. Thus, our review will elucidate how different COC formulations potentially influences exercise performance through their effect on cardiorespiratory and immune changes. This in turn will assist in providing guidance when making decision from the pool of the available oral contraceptives as well as planning future experimental studies to explore how these drugs can be regulated.

## Strength and limitations

- Our review will offer a broad synthesis of the available evidence concerning the relationship between contraceptive use (COC) and cardiorespiratory function, particularly in conjunction with immune activation and metabolic changes.
- Ensuring a comprehensive search strategy will increase the likelihood of identifying and retrieving all relevant articles pertinent to our research questions.
- Our systematic review protocol followed the PRISMA guidelines.
- The potential limitation will centre around the heterogeneity of the available studies in terms of study design, methodological approach, COC formulation and reported outcomes.

## Supporting information

S1 Checklist PRISMA-P checklist. (DOCX)

S1 File Preliminary search strategy. (DOC)
