# Supplementary material for: Effect of combined oral contraceptive on cardiorespiratory function and immune activation in premenopausal women involved in exercise: A systematic review protocol
Source: PLoS One. 2024 Feb 23;19(2):e0298429. doi: 10.1371/journal.pone.0298429 (PMC10889868; doi:10.1371/journal.pone.0298429)
Supplement: S1 File — (DOC) [file pone.0298429.s002.doc]

Supplementary file 2: preliminary search strategy ran on January 11, 2024

| Search | Query | Records retrieved |
| --- | --- | --- |
| #1 | ("contraceptives, oral"[MeSH Terms] OR ("contraceptives"[All Fields] AND "oral"[All Fields]) OR "oral contraceptives"[All Fields] OR ("oral"[All Fields] AND "contraceptives"[All Fields]) OR ("contraception"[MeSH Terms] OR "contraception"[All Fields] OR ("birth"[All Fields] AND "control"[All Fields]) OR "birth control"[All Fields]) OR ("contraceptives oral combined"[Pharmacological Action] OR "contraceptives, oral, combined"[MeSH Terms] OR ("contraceptives"[All Fields] AND "oral"[All Fields] AND "combined"[All Fields]) OR "combined oral contraceptives"[All Fields] OR ("combined"[All Fields] AND "oral"[All Fields] AND "contraceptives"[All Fields])) OR (("contraceptives, oral"[MeSH Terms] OR ("contraceptives"[All Fields] AND "oral"[All Fields]) OR "oral contraceptives"[All Fields] OR ("oral"[All Fields] AND "contraceptive"[All Fields]) OR "oral contraceptive"[All Fields]) AND ("pill s"[All Fields] OR "pills"[All Fields])) OR ("contracept"[All Fields] OR "contracepted"[All Fields] OR "contracepting"[All Fields] OR "contraception"[MeSH Terms] OR "contraception"[All Fields] OR "contraceptions"[All Fields] OR "contraceptive agents"[Pharmacological Action] OR "contraceptive agents"[MeSH Terms] OR ("contraceptive"[All Fields] AND "agents"[All Fields]) OR "contraceptive agents"[All Fields] OR "contraceptives"[All Fields] OR "contraceptive devices"[MeSH Terms] OR ("contraceptive"[All Fields] AND "devices"[All Fields]) OR "contraceptive devices"[All Fields] OR "contraceptive"[All Fields] OR "contraceptive s"[All Fields] OR "contraceptively"[All Fields])) AND (fft[Filter]) | 178,583 |
| #2 | "exercise"[MeSH Terms] OR "exercise"[All Fields] OR "exercises"[All Fields] OR "exercise therapy"[MeSH Terms] OR ("exercise"[All Fields] AND "therapy"[All Fields]) OR "exercise therapy"[All Fields] OR "exercise s"[All Fields] OR "exercised"[All Fields] OR "exerciser"[All Fields] OR "exercisers"[All Fields] OR "exercising"[All Fields] OR ("physical fitness"[MeSH Terms] OR ("physical"[All Fields] AND "fitness"[All Fields]) OR "physical fitness"[All Fields]) OR ("athlete s"[All Fields] OR "athletes"[MeSH Terms] OR "athletes"[All Fields] OR "athlete"[All Fields] OR "athletically"[All Fields] OR "athlets"[All Fields] OR "sports"[MeSH Terms] OR "sports"[All Fields] OR "athletic"[All Fields] OR "athletics"[All Fields]) OR (("heart"[MeSH Terms] OR "heart"[All Fields] OR "cardio"[All Fields]) AND ("workout"[All Fields] OR "workouts"[All Fields])) | 804,474 |
| #3 | "Cardiorespiratory fitness"[MeSH Terms] OR ("cardiorespiratory"[All Fields] AND "fitness"[All Fields]) OR "cardiorespiratory fitness"[All Fields] | 9,857 |
| #3 | ("immune"[All Fields] OR "immuned"[All Fields] OR "immunes"[All Fields] OR "immunisation"[All Fields] OR "vaccination"[MeSH Terms] OR "vaccination"[All Fields] OR "immunization"[All Fields] OR "immunization"[MeSH Terms] OR "immunisations"[All Fields] OR "immunizations"[All Fields] OR "immunise"[All Fields] OR "immunised"[All Fields] OR "immuniser"[All Fields] OR "immunisers"[All Fields] OR "immunising"[All Fields] OR "immunities"[All Fields] OR "immunity"[MeSH Terms] OR "immunity"[All Fields] OR "immunization s"[All Fields] OR "immunize"[All Fields] OR "immunized"[All Fields] OR "immunizer"[All Fields] OR "immunizers"[All Fields] OR "immunizes"[All Fields] OR "immunizing"[All Fields]) AND ("activable"[All Fields] OR "activate"[All Fields] OR "activated"[All Fields] OR "activates"[All Fields] OR "activating"[All Fields] OR "activation"[All Fields] OR "activations"[All Fields] OR "activator"[All Fields] OR "activator s"[All Fields] OR "activators"[All Fields] OR "active"[All Fields] OR "actived"[All Fields] OR "actively"[All Fields] OR "actives"[All Fields] OR "activities"[All Fields] OR "activity s"[All Fields] OR "activitys"[All Fields] OR "motor activity"[MeSH Terms] OR ("motor"[All Fields] AND "activity"[All Fields]) OR "motor activity"[All Fields] OR "activity"[All Fields]) | 569,901 |
| #4 | ("Pre-menopausal"[All Fields] AND ("womans"[All Fields] OR "women"[MeSH Terms] OR "women"[All Fields] OR "woman"[All Fields] OR "women s"[All Fields] OR "womens"[All Fields])) OR (("young"[All Fields] OR "youngs"[All Fields]) AND ("femal"[All Fields] OR "female"[MeSH Terms] OR "female"[All Fields] OR "females"[All Fields] OR "female s"[All Fields] OR "femals"[All Fields]) AND ("adult"[MeSH Terms] OR "adult"[All Fields] OR "adults"[All Fields] OR "adult s"[All Fields])) | 1,023,985 |
| #5 | #1 AND #2 OR #3AND #4  Combination of strings 1, 2, 3 and 4 using the Boolean term “OR”, “AND” | 351 |

There are no database search date limits and no language limits. This is a pilot search strategy carried out on MEDLINE (PubMed) to exemplify the possible strategy of this study. In our final review study, we will include records of the search of all other databases.
